# The use of denosumab in the setting of acute pathological fracture through giant cell tumour of bone

**DOI:** 10.1186/s12957-021-02143-3

**Published:** 2021-02-02

**Authors:** Wolfram Weschenfelder, John M. Abrahams, Luke J. Johnson

**Affiliations:** 1 South Australian Bone and Soft Tissue Tumour Unit, Discipline of Orthopaedic Surgery, Flinders University and Flinders Medical Centre, Adelaide, Australia; 2 Department of Trauma, Hand and Reconstructive Surgery, University Hospital Jena, Am Klinikum 1, D-07747 Jena, Germany

**Keywords:** Distal radius fracture, Giant cell tumour of bone (GCTB), Denosumab, Curettage and grafting

## Abstract

**Background:**

Denosumab (Xgeva^TM^) is a fully human antibody to RANK-Ligand, an important signal mediator in the pathogenesis of giant cell tumour of bone (GCTB). The use of denosumab in the treatment of GCTB has changed the way in which these tumours are managed over the past years.

**Case presentation:**

Described is the case of an acute fracture through a GCTB of the distal radius of a fit and well 32-year-old, non-smoking, female patient following a simple fall onto her outstretched, dominant hand. The aim was to enable joint sparing management for the patient, as opposed to an acute fusion procedure of the carpus. The patient underwent percutaneous k-wire fixation with application of plaster and immediate commencement with denosumab to halt the activity of the GCTB. Bone healing was rapid; plaster and k-wires were removed after 6 weeks. At 6 months denosumab, was ceased and an open curettage and grafting procedure of the tumour bed was undertaken (using MIIG X3, Wright Medical, aqueous calcium sulphate as graft material).

**Conclusions:**

The use of denosumab in the acute setting of pathological fracture through giant cell tumour of bone allowing joint salvage has not been previously described. The treatment was well tolerated and functional outcomes are excellent, with very promising 4-year follow-up.

This novel approach may allow for more joint sparing strategies in the future for other patients in this difficult situation. Further cases will need to be gathered to establish this technique as a suitable treatment pathway.

## Background

The giant cell tumour of bone (GCTB) represents an aggressive lesion, which in 4% of the cases can also have benign pulmonary implants. GCTB accounts for about 6–20% of bone tumours in adults and is mainly diagnosed in the third decade of life [1, 2]. It is typically found in the meta-diaphysis as an osteolytic lesion with eccentric growth (24% distal femur, 23% proximal tibia, 10% distal radius, 6% proximal humerus, 5% distal radius, etc.) [1, 3, 4]. The typical clinical presentation is pain due to mechanical instability, local swelling, decreased range of motion and in approximately 12% of the cases a pathological fracture [3, 4] Histologically, the tumour has three main cellular components: osteoclast-like giant cells, mononuclear spindle-like stromal cells and mononuclear cells of the monocyte/macrophage lineage [2, 5].

The treatment of GCTB depends on localization, extension and classification as per Enneking or Campanacci [4]. It traditionally consists of curettage and grafting with the use of adjuvants for contained defects and en-bloc resection for highly aggressive lesions or where joint salvage is deemed not possible. The main problem lies in the high reported recurrence rate of 20–56% with intralesional treatment and the poorer functional outcome with en-bloc resections [1, 2, 4, 6]. The use of denosumab (Xgeva^TM^) in the treatment of GCTB has changed the way in which these tumours are managed over the past years as it enables a “down-staging” of primarily uncontained defects and ideally creating a salvageable situation, allowing intralesional treatment and joint preservation [2, 7, 8]. Denosumab is a fully human antibody to RANK-Ligand that inhibits the osteoclast-driven bone resorption. In the acute setting following fracture, control of the GCTB is very difficult to manage and previous reports have suggested translocation of the carpus with fusion to the ulna.

The authors are not aware of any previous reports or case series that describe the use of denosumab in the acute setting following a pathological fracture through a GCTB lesion.

## Case presentation

**Fig. 1:**
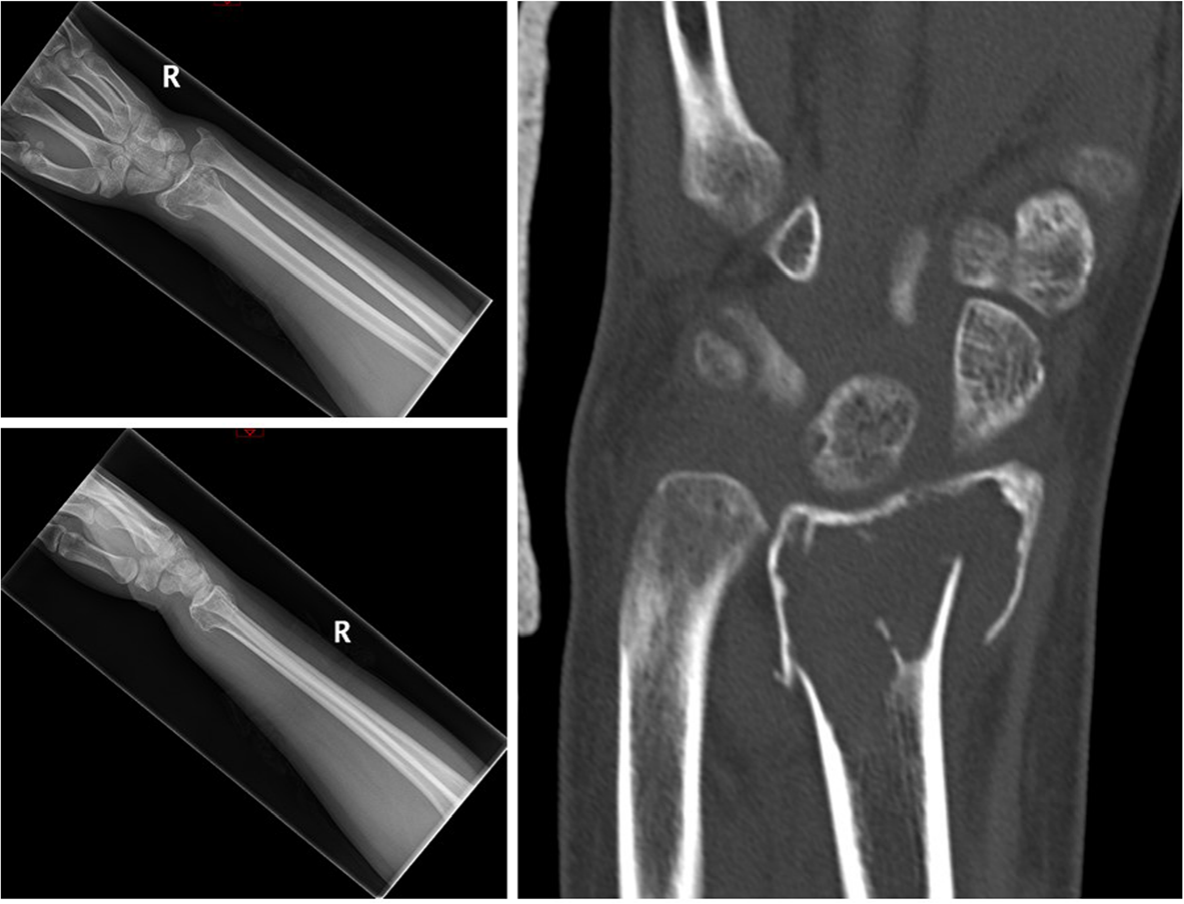
ap and lateral plain radiographs and coronal CT of initial imaging of right wrist

**Fig. 2:**
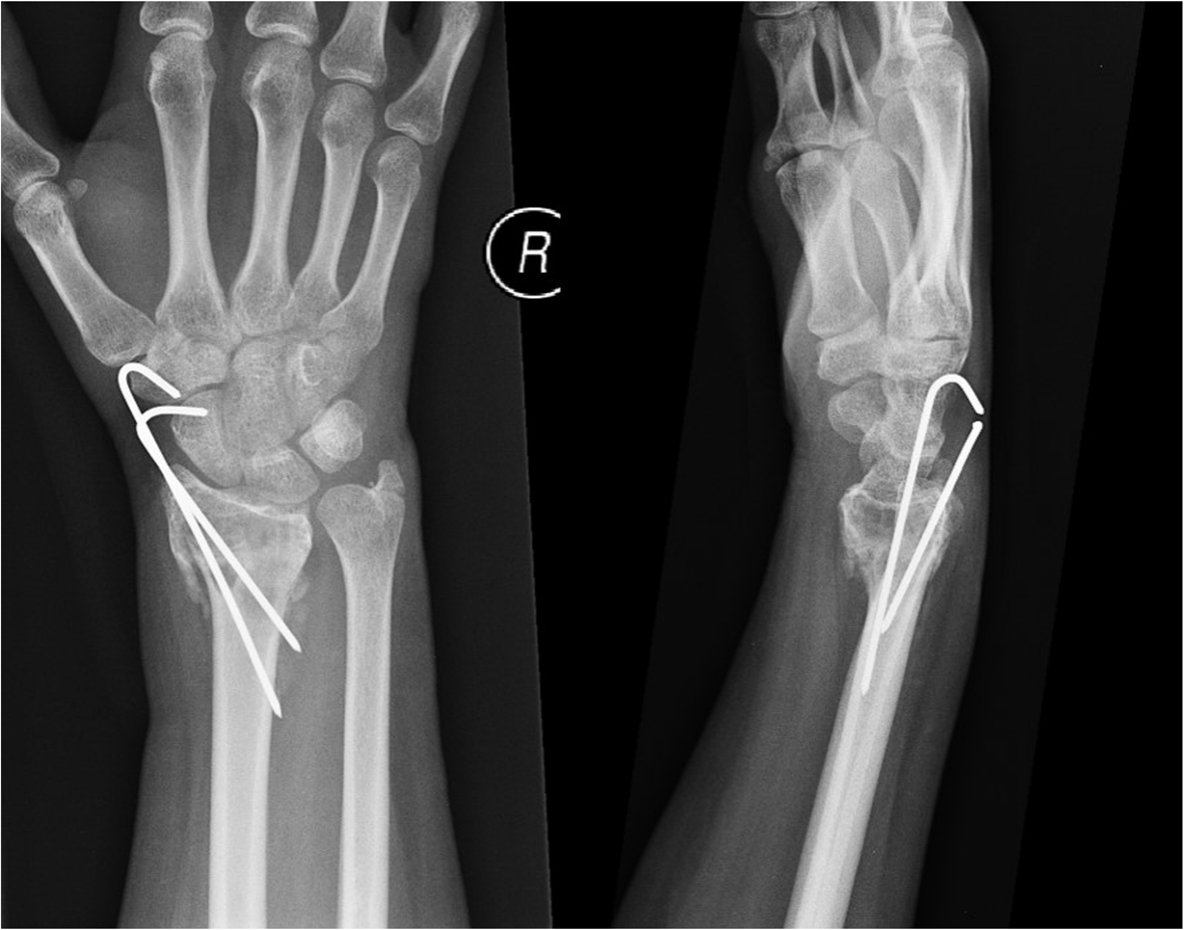
ap and lateral plain radiographs of right wrist 6 weeks after fixation and start of denosumab

**Fig. 3:**
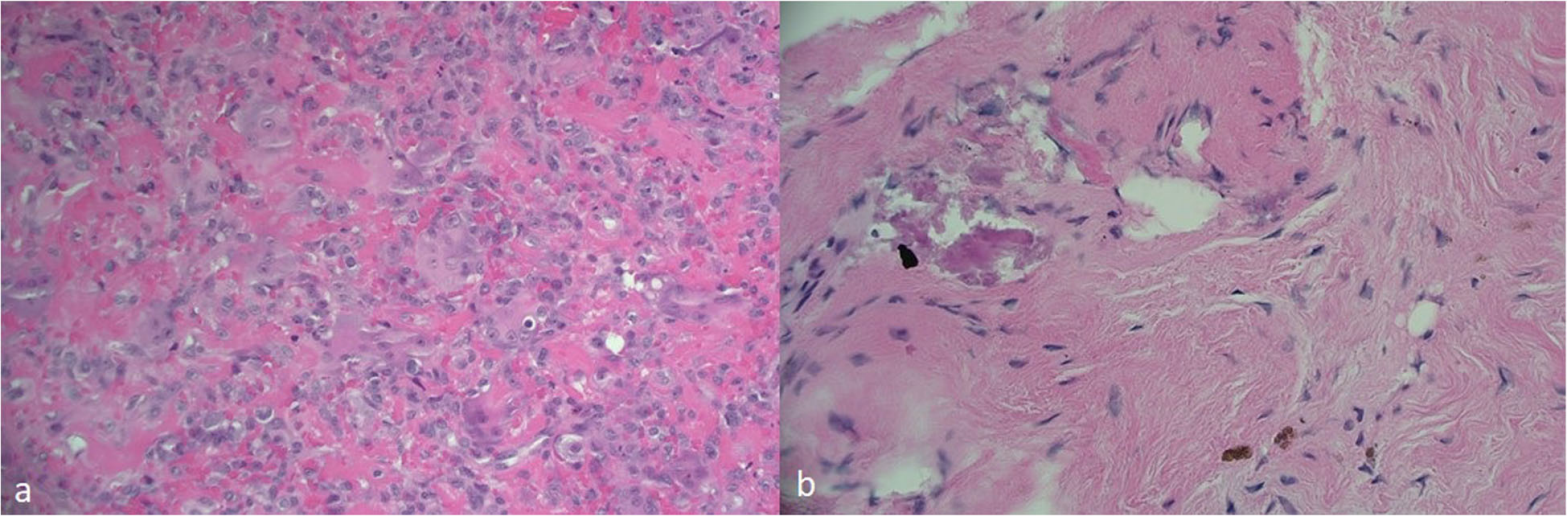
Histologic images. **a** Pre-denosumab—note the large amounts of giant cells on a background of stromal cells. **b** 6 months post-denosumab—no giant cells, bland fibrous infiltrate

Described is the case of acute fracture through a GCTB of the distal radius of a fit and well 32-year-old, non-smoking, female patient following a simple fall onto her outstretched, dominant hand. The aim was for joint salvage of the radio-carpal joint rather than proceeding to an acute fusion procedure. The initial plain radiographs and CT displayed a lytic bone lesion at the distal end of the radius and subsequent open biopsy on day 1 confirmed a GCTB lesion (see Fig. 1). On MRI, the lesion was scalloping and thinning the cortex but did not show a soft-tissue involvement outside the fracture side. Furthermore, the articular surface seemed to be intact. Based on the imaging, the lesion was staged as a Campanacci type II. After multidisciplinary team discussion and informed consent, the patient underwent percutaneous k-wire fixation on day 7, application of plaster and immediate commencement with denosumab to halt the activity of the GCTB (120 mg every 4 weeks). Plaster and k-wires were removed at the 6 week mark postoperatively. Bone healing was rapid (see Fig. 2). Tumour inactivity and bone reconstitution was confirmed with serial radiographs. At 6 months denosumab was ceased and an open curettage and grafting procedure of the tumour bed was undertaken using a dorsal approach and aqueous calcium sulphate as graft material. The histology taken at that time showed a very good response to the denosumab treatment (see Fig. 3).

**Fig. 4:**
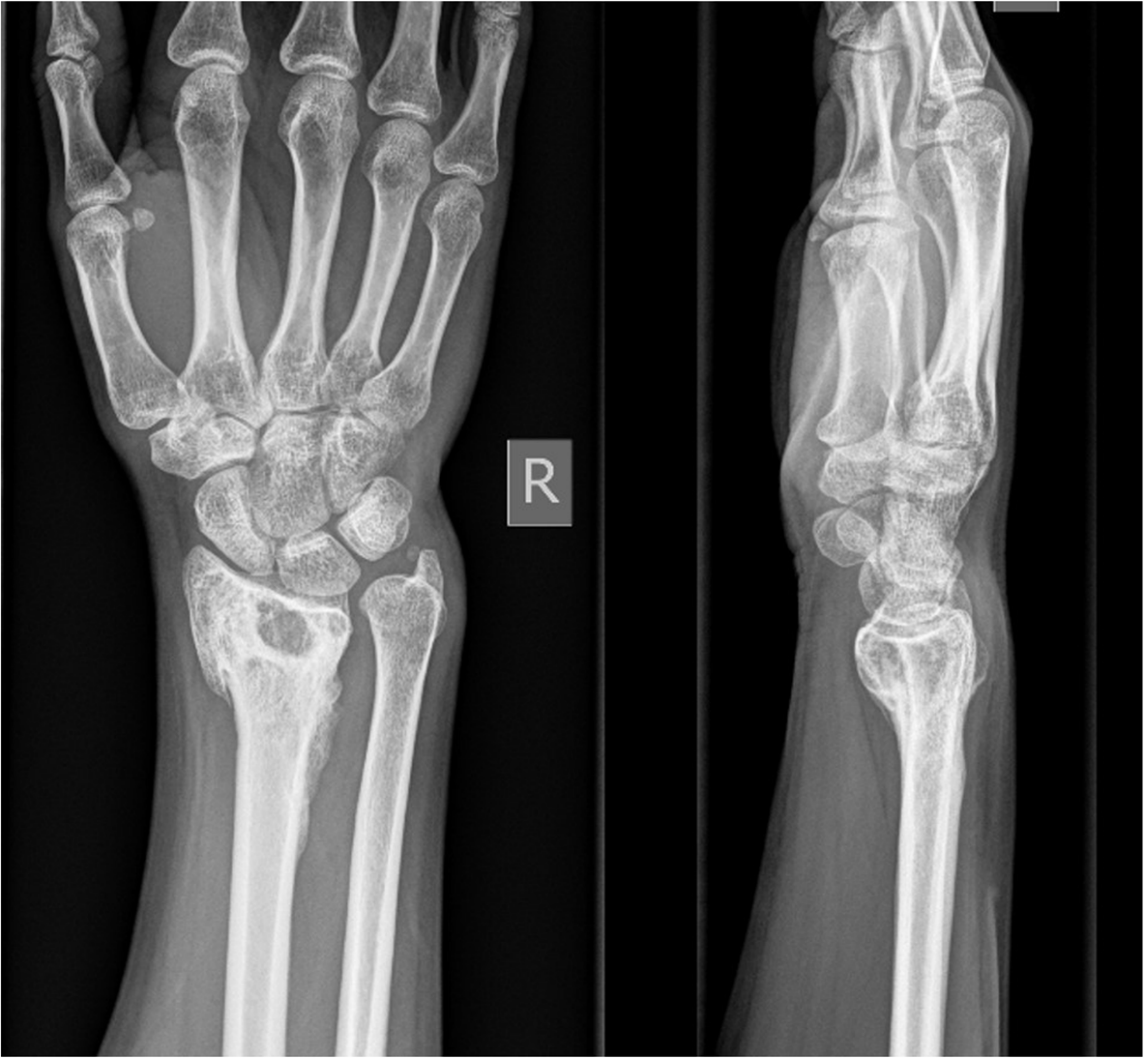
ap and lateral plain radiographs of right wrist 4 years after treatment

The patient tolerated the denosumab therapy with no reported side effects. Excellent functional restoration, with near full range of motion. Toronto Extremity Salvage Score (TESS) at 1 year was 98, at 4 years 99. Visual analogue score = 1 with heavy activity; 0 at rest. No evidence of local recurrence at 4-year post-treatment on X-ray (see Fig. 4).

## Discussion and conclusions

The use of denosumab to downstage GCTB has been introduced in the last decade [1, 9, 10] and has shown promising results allowing more joint preserving therapies and treatment in cases of unresectable tumour locations. Van Langevelde et al. documented the positive effects of denosumab treatment on imaging with development of a new sclerotic neocortex and matrix osteosclerosis on plain X-rays. The articular surface and subarticular bone—which are considered the most important predictors for joint salvage—are best evaluated on CT [11]. There exist standardised protocols for the neoadjuvant treatment with denosumab with a duration between 3 and 6 months [7, 9, 10]. The timing and influence of a neoadjuvant treatment with denosumab on recurrence of GCTB is still under investigation. Chen et al. reviewed 10 studies with a total of 1082 cases and found an increased risk of local recurrence after preoperative treatment of GCTB with denosumab [12]. However, in our case, the aim of the denosumab treatment was the downstaging of the tumour to facilitate a joint salvage procedure and therefore a preoperative treatment was imperative.

Denosumab as an inhibitor of osteoclast-driven bone resorption does not impair fracture healing, in contrast animal studies showed that callus volume and torsional rigidity are increased. Additionally the FREEDOM trial did not show delayed healing or non-union under denosumab treatment. This was a large double-blind placebo-controlled study consisting of 7808 women with a total of 851 non-vertebral fractures during the study period [13].

However, there are side effects related to the use of denosumab, e.g., pain, hypocalcemia, osteonecrosis of the jaw, urinary tract infections, and pathological femur fractures that need to be discussed with the patient before starting the treatment. Additionally, there have been case reports of a potential association of the treatment of a GCTB with denosumab and sarcomatous transformation. Due to the extremely low numbers of only 11 cases worldwide until December 2018, the causal association is unclear [14].

The fracture healing was considerable fast in our described case and there were no side effects of the denosumab treatment. The defect after curettage was grafted with aqueous calcium sulphate. Johnson and Clayer demonstrated good clinical results and bone remodelling using this method for contained defects of bone after curettage procedures avoiding the side effects and potential complications of autologous bone grafting [15]. The final X-ray after 4 years showed an almost entirely remodelled distal radius with intact articular surface and no signs of a tumour recurrence.

The use of denosumab in the acute setting of pathological fracture through giant cell tumour of bone allows joint salvage has not been previously described. The treatment was well tolerated and functional outcomes are excellent. This novel approach may allow for more joint sparing strategies in the future for other patients in this difficult situation. Further cases will need to be gathered to establish this technique as a suitable treatment method.

## Data Availability

Not applicable.

## Abbreviations

GCTB: Giant cell tumour of bone
TESS: Toronto extremity salvage score
